# A Risk-Factor Guided Approach to Reducing Lactic Acidosis and Hyperlactatemia in Patients on Antiretroviral Therapy

**DOI:** 10.1371/journal.pone.0018736

**Published:** 2011-04-11

**Authors:** Lynn T. Matthews, Janet Giddy, Musie Ghebremichael, Jane Hampton, Anthony J. Guarino, Aba Ewusi, Emma Carver, Karen Axten, Meghan C. Geary, Rajesh T. Gandhi, David R. Bangsberg

**Affiliations:** 1 Division of Infectious Disease, Beth Israel Deaconess Medical Center, Boston, Massachusetts, United States of America; 2 HIV Program, McCord Hospital, Durban, South Africa; 3 Ragon Institute of Massachusetts General Hospital, Massachusetts Institute of Technology, and Harvard University, Boston, Massachusetts, United States of America; 4 Institute of Health Professions, Massachusetts General Hospital, Boston, Massachusetts, United States of America; 5 Division of Internal Medicine, Harvard Vanguard Medical Associates, Boston, Massachusetts, United States of America; 6 Department of Emergency Medicine, University Hospital of Wales, Cardiff, Wales; 7 Albany Medical College, Albany, New York, United States of America; 8 Division of Infectious Disease, Massachusetts General Hospital, Boston, Massachusetts, United States of America; 9 Mbarara University of Science and Technology, Mbarara, Uganda; 10 Center for Global Health, Massachusetts General Hospital, Boston, Massachusetts, United States of America; University of Cape Town, South Africa

## Abstract

**Background:**

Stavudine continues to be used in antiretroviral treatment (ART) regimens in many resource-limited settings. The use of zidovudine instead of stavudine in higher-risk patients to reduce the likelihood of lactic acidosis and hyperlactatemia (LAHL) has not been examined.

**Methods:**

Antiretroviral-naïve, HIV-infected adults initiating ART between 2004 and 2007 were divided into cohorts of those initiated on stavudine- or zidovudine-containing therapy. We evaluated stavudine or zidovudine use, age, sex, body mass index (BMI), baseline CD4 cell count, creatinine, hemoglobin, alanine aminotransferase, and albumin as predictors of time to LAHL with Cox Proportional Hazards (PH) regression models.

**Results:**

Among 2062 patients contributing 2747 patient years (PY), the combined incidence of LAHL was 3.2/100 PY in those initiating stavudine- and 0.34/100 PY in those initiating zidovudine-containing ART (RR 9.26, 95% CI: 1.28–66.93). In multivariable Cox PH analysis, stavudine exposure (HR 14.31, 95% CI: 5.79–35.30), female sex (HR 3.41, 95% CI: 1.89–6.19), higher BMI (HR 3.21, 95% CI: 2.16–4.77), higher creatinine (1.63, 95% CI: 1.12–2.36), higher albumin (HR 1.04, 95% CI: 1.01–1.07), and lower CD4 cell count (HR 0.96, 95% CI: 0.92–1.0) at baseline were associated with higher LAHL rates. Among participants who started on stavudine, switching to zidovudine was associated with lower LAHL rates (HR 0.15, 95% CI: 0.06–0.35). Subgroup analysis limited to women with higher BMI≥25 kg/m2 initiated on stavudine also showed that switch to zidovudine was protective when controlling for other risk factors (HR 0.21, 95% CI .07–0.64).

**Conclusions:**

Stavudine exposure, female sex, and higher BMI are strong, independent predictors for developing LAHL. Patients with risk factors for lactic acidosis have less LAHL while on zidovudine- rather than stavudine-containing ART. Switching patients from stavudine to zidovudine is protective. Countries continuing to use stavudine should avoid this drug in women and patients with higher BMI.

## Introduction

Lactic acidosis is a potentially fatal side effect of nucleoside analog reverse transcriptase inhibitors (NRTIs) [1], [2], which are commonly used in combination antiretroviral therapy (ART). This complication is related to NRTI-induced mitochondrial toxicity possibly due to structural similarities between mitochondrial DNA polymerase and HIV-reverse transcriptase (the target of NRTIs) [3]. The incidence of lactic acidosis among patients on ART ranges from 1–4 per 100 patient years in resource-rich settings and is as high as 10 per 100 patient years in sub-Saharan African cohorts [4], [5], [6], [7], [8], [9], [10], [11]. The lactic acidosis case-fatality rate in resource-limited settings can be as high as 60% [12].

Of the NRTIs, the dideoxynucleosides (stavudine and didanosine) confer the highest risk of lactic acidosis [1], [2], [5]. While stavudine is rarely used in resource-rich settings and is no longer recommended by the World Health Organization for initial treatment of HIV-1 infection [13], it remains an important component of standard ART regimens in many resource-limited countries, largely due to cost [14], [15]. In South Africa where stavudine is no longer recommended for use in first-line therapy, patients receiving stavudine-containing ART are only switched if there is evidence of toxicity, again because of financial constraints. In settings where stavudine is widely prescribed, lactic acidosis is a frequent cause of morbidity and mortality [1], [2], [4], [5], [6], [7], [8], [9], [10], [16] and is associated with high losses to follow-up and treatment discontinuation [15].

Observational studies suggest that specific risk factors associated with the development of hyperlactatemia include female sex [1], [4], [7], [11], [16], [17], [18], elevated weight or body-mass index (BMI) [1], [11], [16], [17], [18], older age (>40 years) [1], [11], and lower CD4 cell counts [1]. Where financial constraints prevent comprehensive adoption of less-toxic agents, a risk factor-guided approach to choosing an initial regimen may reduce the incidence of lactic acidosis. Studies have shown that after resolution of lactic acidosis it is safe to treat patients with zidovudine (an alternative thymidine analog NRTI which is widely used in resource-limited settings) [10], [19], but none have examined whether avoiding stavudine in patients with lactic acidosis risk factors reduces incidence of lactic acidosis or hyperlactatemia.

Until April 2010, first-line therapy in South Africa included stavudine, lamivudine, and either efavirenz or nevirapine. Based on observational findings from a site-specific study that identified a high incidence of lactic acidosis in women with BMI≥28 kg/m^2^, in August 2005 the HIV Clinic at McCord Hospital in Durban, South Africa substituted zidovudine for stavudine in initial ART for patients with these two risk factors [7]. The policy continued until March 2007, when the clinic was accredited as a Department of Health site and required to follow Department of Health guidelines for ART, including the use of stavudine as part of initial regimens.

To evaluate the impact of risk factor-guided selection of initial therapy, we compared the combined incidence of lactic acidosis and hyperlactatemia among treatment-naive patients initiating stavudine-containing therapy with those starting zidovudine-containing therapy. We hypothesized that risk-factor-guided ART (initiating women with BMI≥28 kg/m2 on zidovudine rather than stavudine) would be associated with decreased incidence of lactic acidosis and hyperlactatemia. We also assessed predictors of lactic acidosis and hyperlactatemia.

## Methods

### Ethics statement

Ethics approvals were obtained from the McCord Hospital Medical Ethics Research Committee and from the Partners Healthcare Institutional Review Board (Boston, MA). Given the nature of the study (retrospective chart review), the requirement for informed consent was waived by the ethics committees.

### Study design and population

Patient data were collected from the outpatient HIV clinic at McCord Hospital in Durban, South Africa which has initiated over 8000 patients on ART. During the study period, initial ART included two NRTIs and one NNRTI: stavudine (30 mg twice daily; 40 mg twice daily if weight >60 kg) or zidovudine plus lamivudine and either efavirenz or nevirapine.

The study population included antiretroviral (ARV)-naïve, HIV-infected adults (age ≥18 years) with baseline laboratory data and at least one follow-up visit after ART initiation. Two retrospective cohorts were identified. The first cohort included patients who initiated stavudine-containing therapy between July 2004 and March 2007. The second cohort included patients who initiated zidovudine-containing ART between July 2004 and March 2007. Both cohorts included patients who initiated ART between August 2005 and March 2007 when the clinic made women with BMI≥28 kg/m2 eligible for initiation of zidovudine -containing therapy or for regimen switch from stavudine to zidovudine.

### Outcomes and their measurement

The primary outcome was event-free survival defined as the time from treatment initiation to development of lactic acidosis (symptomatic or asymptomatic) or hyperlactatemia (symptomatic or asymptomatic) (Table 1). Lactic acidosis and hyperlactatemia were defined based on AIDS Clinical Trials Group criteria [20]. Lactic acidosis is defined as having a lactate level above the upper limit of normal (4.4 mmol/L) along with evidence of acidosis (bicarbonate level <20 mmol/L or pH<7.35). Hyperlactatemia is defined as a lactate level greater than the upper limit of normal without evidence of acidosis. Cases of symptomatic lactic acidosis or hyperlactatemia met the above criteria and had new, otherwise unexplained symptoms of nausea, vomiting; abdominal pain, discomfort, or distention; increased hepatic transaminases; fatigue; dyspnea; weight loss (≥5%); or muscle weakness. Because these patients were ambulatory and often did not have repeat measurements, confirmed elevation of lactate levels was not required if at least two symptoms were present.

**Table 1:** Criteria for lactic acidosis and hyperlactatemia outcomes^1^.

	Asymptomatic lactic acidosis	Symptomatic lactic acidosis	Asymptomatic hyperlactatemia	Symptomatic hyperlactatemia
Lactate (mmol/L)	≥4.4	≥4.4	≥4.4	≥4.4
Abnormal values required	≥2	≥1	≥2	≥1
Acidosis^2^	+	+	−	−
Symptoms^3^	−	+	−	+

1 Based on AACTG criteria [19].

2 Bicarbonate <20 mmol/L or pH<7.35.

3 New or otherwise unexplained symptoms of nausea or vomiting, abdominal pain or discomfort, abdominal distention, increased hepatic transaminases, unexplained fatigue, dyspnea, weight loss (≥5%), or muscle weakness.

Blood was drawn for lactate levels without use of a tourniquet and specimens were transported on ice and processed within four hours (Beckman Coulter, Synchron systems, California, USA). A handheld lactate detection device, a reliable proxy for serum samples, was introduced in 2006 (Accutrend model #3012522) [21], [22] and was used for initial screening in addition to serum lactate testing.

Outcomes were classified from a review of the medical records of patients initiating ART during the study period. This review was facilitated by the requirement that clinicians record the reason for any change or discontinuation in ARV regimen from an electronic pull down menu. For patients who had a regimen change noted in the electronic record, paper charts were reviewed for the following: 1) documentation of a regimen change due to lactic acidosis; 2) documentation of a regimen change and signs or symptoms that could be consistent with lactic acidosis or hyperlactatemia (nausea, vomiting, abdominal discomfort, bloating, increased hepatic transaminases, fatigue, dyspnea, weight loss, muscle weakness); 3) documentation of a regimen change without specific reason listed; 4) death. Serum lactate test results for all study patients were reviewed. A lactate value above 3 mmol/L prompted review of the medical record for symptoms of hyperlactatemia, all available lactate values, other possible causes for symptoms or elevated lactate levels, and clinical outcome. Data were abstracted using standardized abstraction forms (LM, AE, JH). For a subset of patients (n = 20), two physicians carried out the abstractions with 100% agreement on outcome classification (JH, LM). Cases with unclear outcomes were adjudicated by a senior clinician (RG).

We also identified patients for whom clinicians had changed ART due to peripheral neuropathy, lipodystrophy, high BMI, and drug resistance, as indicated in the electronic medical record. We identified patients with regimen switch for clinical suspicion of LA or HL but who did not meet criteria. These subjects were not censored at change in regimen but followed out to a total two years of follow-up from treatment initiation. In addition, we identified subjects who changed clinic site, stopped ART, died or were lost to follow up.

### Covariates

Covariates were obtained from paper chart abstractions and included weight at treatment initiation (within 3 months) and height. Weights obtained during pregnancy were excluded. BMI was calculated (kg/m^2^) for all subjects in whom height and baseline weight were available. Sex, date of birth and baseline (the last value prior to ART initiation, or within 2 weeks) CD4 count, creatinine, hemoglobin, alanine aminotransferase, and albumin were extracted from the electronic record or the paper chart. All specimens were processed using standardized methods at laboratories in Durban.

### Time to event or censor

The primary outcome was 2-year event-free survival (EFS) defined as the time from treatment initiation up to development of lactic acidosis or hyperlactatemia. Patients were also censored for loss to follow-up, change in clinic site, termination of treatment, death, or at study end. All others were followed for two years or until the primary outcome. Time on stavudine and zidovudine was calculated from start and stop dates entered by clinicians in the medical record.

### Analysis

We calculated crude incidence rates for the combined primary outcome (LAHL), the combined incidence of peripheral neuropathy and lipodystrophy, death, and loss-to-follow-up. Confidence intervals for event rates based on initial therapy with stavudine or zidovudine were estimated using methods for exact binomial confidence intervals and compared using Chi-square tests [23]. Kaplan-Meier curves were plotted for event-free survival based on initial treatment and rates were compared using the log-rank test statistic. Univariate and multivariate analyses using Cox proportional hazards (PH) regression models were utilized to assess the effect of treatment on time to event [24]. We evaluated time on zidovudine or stavudine as a time-varying covariate to account for variable time on drug among patients whose regimens were switched in the absence of the outcome of interest (e.g. switch for peripheral neuropathy or increased BMI). Covariates for multivariate analysis were selected based on significance (p value<0.05) in univariate analysis and significant covariates in the literature. CD4 count was modeled as a continuous variable with the effect size reported per 10-cell increment. BMI was modeled on a natural logarithmic scale with effect size reported per 30% shift. In the full model, BMI deviated from the proportional hazards assumption and was modeled with a time-dependent association for early (within the first year) and late (after one year) failure. In subgroup analysis, BMI followed proportional hazards. All statistical analyses were carried out using SAS version 9.2 for Windows.

## Results

### Baseline patient and disease characteristics

Two-thousand-sixty-two patients contributing 2747 person years of follow-up were included in the study. The median age was 34.7 years (IQR 29.8, 40.6) and 60% were women. Eighty-nine percent initiated therapy with a stavudine-containing regimen. One-hundred sixty one (77%) of those who were initiated on a zidovudine-containing regimen were started because of higher BMI or other perceived lactic acidosis risk factors. The remaining patients were initiated on zidovudine because of pre-existing lipodystrophy (<1%), peripheral neuropathy (<1%), pregnancy (10%), or unknown reason (10%).

Median CD4 count at entry was 80 cells/mm3 (IQR 29–142). Median BMI for subjects with complete data (88% had documented weight at entry, 76% had documented height) was 22 kg/m2 (IQR 20, 26). Compared with those initiated on a stavudine-containing regimen, patients started on zidovudine were older, more likely to be female, had a higher BMI, higher CD4 cell count, higher albumin and higher hemoglobin. Other characteristics are described in Table 2.

**Table 2:** Patient characteristics at study entry by treatment arm.

	Initial ART includes:		
Variable	Stavudine	Zidovudine	p-value^1^
Number (patient years follow-up)	1853 (2460)	209 (287)	
Age, years *Mean (SD)*	35.7 (8.3)	37.8 (9.6)	<.001
Patient years of follow up *Mean (SD)*	1.3 (0.7)	1.4 (0.6)	<.001
Female *n (%)*	1078 (58.2)	188 (90)	<.001
BMI (kg/m^2^) *Median (IQR)*	22 (19, 24)	30 (28, 33)	<.001
CD4 (cells/mm^3^) *Median (IQR)*	75 (27, 138)	129 (61, 172)	<.001
Creatinine (mg/dL) *Median (IQR)*	1.0 (0.4)	0.9 (0.3)	.21
ALT (IU/L) *Median (IQR)*	24 (18, 35)	23 (17, 32)	.27
Albumin (g/L) *Median (IQR)*	31.2 (7.3)	34.9 (5.3)	<.001
Hemoglobin (g/dL) *Median (IQR)*	10.8 (2.1)	11.6 (1.3)	<.001

1 Chi-square test was used for categorical variables, T-test for continuous where mean and standard deviation reported, and Wilcoxon rank sum where median and IQR reported.

### Outcomes for full cohort

In intention to treat analysis, combined incidence of LAHL was 3.2/100 PY in the stavudine- and 0.34/100 PY in the zidovudine-initiated group (RR 9.26, 95% CI 1.28–66.93, p = .007). There were 36 lactic acidosis and 43 hyperlactatemia events in the stavudine group. In contrast, there was 1 lactic acidosis event in the zidovudine group: this occurred in a woman who initiated zidovudine-based therapy because of high BMI (31 kg/m2); one year later, she was switched to stavudine because of anemia; after eight months on stavudine-containing ART, she was diagnosed with lactic acidosis. Mortality due to causes other than LAHL was 8.3% and 2.8% in stavudine- and zidovudine-initiated patients, respectively (RR = 2.89, 95% CI:1.45–5.78, p = 0.001). The combined incidence of physician-reported peripheral neuropathy and lipodystrophy was 16.8/100 PY in stavudine- and 0.34/100 PY in zidovudine-initiated groups (RR = 59.84, 95% CI: 8.36–428.12, p<0.001). Loss to follow-up was equivalent between the two groups (RR = 1.42, 95% CI: 0.68–2.96, p = 0.35). (Table 3)

**Table 3:** Incidence of mitochondrial toxicity, death and loss to follow-up by initial treatment.

	Initial ART includes:			
Outcome	Stavudine (Incidence/100 PY)	Zidovudine (Incidence/100 PY)	Relative Risk Ratio [95% CI]	Chi-Square p-value
Lactic acidosis or hyperlactatemia^1^	79 (3.2)	1 (0.3)	9.26 [1.28, 66.93]	.007
Mortality due to cause other than LAHL	205 (8.3)	8(2.8)	2.89 [1.45, 5.78]	.001
Peripheral neuropathy or lipodystrophy^2^	414 (16.8)	1 (0.3)	59.84 [8.36, 428.12]	<.001
Loss to follow-up	99 (4.0)	8 (2.8)	1.42 [0.68, 2.96]	.35

1 Primary endpoint: 37 lactic acidosis, 43 hyperlactatemia.

2 As indicated by clinician report in the medical record.

**Figure 1:**
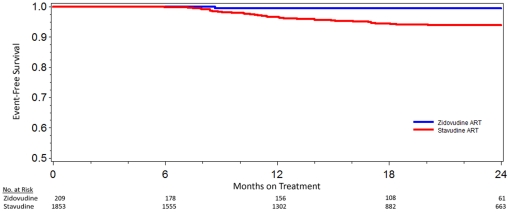
Kaplan Meier curves for lactic acidosis/hyperlactatemia-free survival for patients initiated on either stavudine- or zidovudine-containing antiretroviral therapy (p = .006).

In univariate Cox proportional hazards analysis, stavudine in the initial treatment regimen, female sex, higher BMI, and higher baseline albumin were each associated with increased risk of LAHL (Table 4). The Kaplan Meier curve for time to LAHL based on initial treatment regimen is shown in Figure 1 (p = .006).

**Table 4:** Univariate and multivariate Cox Regression Analysis for time to lactic acidosis or hyperlactatemia.

Variable	Hazards Ratio [95% CI]	p-value	Adjusted Hazards Ratio [95% CI]	p-value
Age (years)	1.02 [0.99, 1.04]	.14	–	.22
Female sex	2.22 [1.31, 3.75]	.003	**3.42 [1.89, 6.19]**	**<.0001**
BMI in first year (30% change kg/m^2^)	1.53 [1.10, 2.12]	.01	**3.21 [2.16, 4.77]**	**<.0001**
BMI after first year (30% change kg/m^2^)	0.76 [0.44, 1.32]	.33	–	.55
Stavudine use	5.81 [2.52, 13.43]	<.0001	**14.31 [5.79, 35.30]**	**<.0001**
Initial CD4 count (10 cells/mm^3^)	0.99 [0.96, 1.03]	.80	**0.96 [0.92, 1.00]**	**.04**
Initial Albumin (g/L)	1.04 [1.01, 1.07]	.004	**1.04 [1.01, 1.07]**	**.004**
Initial Creatinine (mg/dL)	1.00 [0.99, 1.01]	.09	**1.63 [1.12, 2.36]**	**.010**
Initial ALT (IU/L)	1.00 [0.99, 1.01]	.38	–	–
Hemoglobin (g/dL)	1.07 [0.95, 1.19]	.26	–	–

Multivariate model with 80 events, 1546 subjects with complete data for all variables.

In multivariable Cox PH regression to assess predictors of event-free survival, hemoglobin and ALT were removed but age and CD4 cell count were included because prior data and *a priori* knowledge suggested an association with lactic acidosis [1]. Creatinine was into the full model when it was found to be significant in subgroup analysis. The adjusted hazards of experiencing LAHL was higher for those on stavudine (HR = 14.31, 95% CI 5.79–35.30), women (HR = 3.41, 95% CI: 1.89–6.19), subjects with higher BMI in the first year (HR = 3.21, 95% CI: 2.16–4.77), higher albumin (HR = 1.04, 95% CI:1.01–1.07), higher creatinine (HR = 1.63, 95% CI 1.12–2.36), or lower baseline CD4 cell count (HR = 0.96, 95% CI: 0.92–1.00) at baseline (Table 5). Among those initiated on stavudine, the hazards of experiencing LAHL was lower for those who were switched to zidovudine during follow-up (HR 0.15, 95% CI 0.06–0.35).

**Table 5:** Patient characteristics at study entry by treatment arm, limited to women with BMI≥25 kg/m^2^.

	Initial ART includes:		
Variable	Stavudine	Zidovudine	p-value^1^
Number (patient years follow-up)	194 (274)	132 (190)	
Age (years) *Mean (SD)*	36 (7)	38 (9)	.03
Patient years of follow up *Mean (SD)*	1.4 (0.6)	1.4 (0.6)	.64
BMI (kg/m^2^) *Median (IQR)*	27 (26, 30)	30 (29, 34)	<.0001
CD4 (cells/mm^3^) *Median (IQR)*	99 (64)	122 (58)	.0003
Creatinine (mg/dL) *Median (IQR)*	0.85 (0.77, 0.94)	0.88 (0.80, 0.98)	.02
ALT (IU/L) *Median (IQR)*	22 (17, 32)	22 (17, 30)	.97
Albumin (g/L) *Median (IQR)*	32 (6)	36 (4)	<.0001
Hemoglobin (g/dL) *Median (IQR)*	11.1 (1.7)	11.7 (1.1)	.0001

1 T-test for continuous where mean and standard deviation reported, and Wilcoxon rank sum where median and IQR reported.

### Outcomes for women with higher BMI

Women with BMI greater than or equal to 25 kg/m2 comprised 326 patients with 434 years of follow-up. The 194 women initiated on stavudine were younger; with lower BMI, baseline CD4 cell count, creatinine, albumin, and hemoglobin compared with 132 women initiated on zidovudine (Table 5). Obese women initiated on stavudine had 22 LAHL events (8.0/100 woman years), compared with 1 (0.53/100 woman years) among those initiated on zidovudine (RR = 9.94, 95% CI, 1.46–67.91, p = .0002). When controlling for BMI, CD4 cell count, albumin, creatinine, and age, stavudine use was associated with a 13-fold increase in hazards of LAHL (HR 13.37, 95% CI 4.31–41.53) (Table 6). For women in this subgroup who initiated on stavudine-containing therapy, switching to zidovudine was protective (HR 0.21, 95% CI 0.07–0.64, p = 0.006).

**Table 6:** Multivariate Cox Regression Analysis for time to lactic acidosis or hyperlactatemia limited to women with BMI≥25 kg/m2.

Variable	Adjusted Hazards Ratio [95% CI]	p-value
BMI (30% change kg/m^2^)	**3.15 [1.39, 7.17]**	**.005**
Stavudine use	**13.37 [4.31, 41.53]**	**<.0001**
Initial CD4 (10 cells/mm^3^)	–	0.99
Initial Albumin (g/L)	1.01 [0.94, 1.08]	0.83
Initial Creatinine (mg/dL)	**1.75 [1.17, 2.62]**	**.006**

Multivariate model with 20 events, 298 subjects with complete data for all variables.

Of the 194 women with higher BMI who initiated stavudine-inclusive therapy, 137 were switched to zidovudine for reasons other than LAHL. Baseline characteristics (age, BMI, CD4 cell count, creatinine albumin, ALT, hemoglobin) were not significantly different from women with higher BMI initiated on stavudine-treatment who did not switch treatment arms. Women were switched for high BMI (79, 56%); lipodystrophy, peripheral neuropathy, or these plus elevated BMI (47, 34%); lab values and/or symptoms suggestive of hyperlactatemia that did not meet criteria for LAHL (7, 5%); and the remainder were switched for anemia, pregnancy, rash or other reasons. These participants subsequently contributed an additional 131.7 woman-years of follow-up (mean 1.1 years ±0.5) during which there were 5 LAHL events (3.8/100 woman years). All but one event occurred within 2–8 weeks of switching off stavudine after an average of 0.6±0.4 years on stavudine, suggesting that the recent and cumulative stavudine exposure contributed to the toxicity. When controlling for other LAHL risk factors, switch to zidovudine conferred 80% lower hazards of LAHL for this subgroup (HR 0.21, 95% CI 0.07–0.64, p = .006). The remainder of women in this subgroup of obese women, initiated on stavudine and switched to zidovudine, included two who subsequently had anemia and two who died; the rest were followed until the end of the study, change in service provider, or a maximum of two years of follow-up without adverse events.

## Discussion

In our study of 2062 HIV-positive patients who initiated ART, stavudine use confers a fourteen-fold increased risk of developing hyperlactatemia or lactic acidosis when controlling for other risk factors (HR 14.31, 95% CI 5.79–35.30). Other risk factors for the primary outcome of LAHL were female sex, higher baseline BMI, higher baseline creatinine or albumin, and lower initial CD4 cell count. For patients who started a stavudine-containing regimen, switching to zidovudine was associated with 85% lower hazards of developing LAHL (HR 0.15, 95% CI 0.06–0.35). For the high-risk subgroup of women with BMI≥25 kg/m2 who initiated therapy on stavudine-containing ART, switch to zidovudine was also protective when controlling for other risk factors (HR 0.21, 95% CI 0.07–0.64).

Our study adds to the literature by demonstrating that female sex is a strong independent risk factor for developing LAHL [1], [4], [7], [11], [16], [17], [18]. Higher weight has been associated with these outcomes in prior studies, but this is the first to confirm a relationship with BMI and LAHL when controlling for other covariates [1], [11], [16], [17], [18]. For every 30% change in BMI (i.e. 18 to 23 kg/m^2^ or 24 to 31 kg/m^2^), we observed a three-fold increase in the LAHL rate (HR = 3.21, 95% CI: 2.16–4.77). For the full dataset, the effect was only significant in the first year of follow-up which may reflect increased risk earlier in treatment or insufficient power to detect an association after the first year. During the study, patients with weight >60 kg received 80 mg of stavudine daily, which has been linked to worse mitochondrial toxicities compared to use of 60 mg [25]. We were unable to control for stavudine dose; thus, the high incidence of LAHL in patients with higher BMI might be related to higher stavudine dose. This possibility is supported by observations that patients on higher dose stavudine (40 mg twice daily) have a higher incidence of elevated lactate than those who receive lower doses (20 or 30 mg twice daily) [16], [25]. However, given that multiple studies involving varying stavudine dose have found an association between higher weight or BMI and lactic acidosis [7], [11], [17] while, in some cases, controlling for dose [16], [18], it is unlikely that drug dosing explains the entire effect. Furthermore, a three-fold increase in hazards of LAHL was observed in our subgroup analysis of women with BMI≥25 kg/m2 (who likely received uniform stavudine dosing).

Higher creatinine was associated with increased hazards of LAHL, about 25% per 1 mg/dL unit increase in creatinine. This risk factor has not been reported in prior univariate analyses and has not been included in studies that control for other risk factors, but is not unexpected given the kidney's role in lactate metabolism [26]. We also found that higher albumin is associated with an increased risk of LAHL and a small protective effect of higher CD4 cell count at treatment initiation (4% decrease in hazards for each 10-point increase in baseline CD4 cell count). Two other studies have also found an association between CD4 cell count and lactic acidosis [1], [27]. Each of these associations (CD4 cell count, creatinine and albumin) was small with confidence intervals close to one.

The Lactic Acidosis International Study group showed an association of older age (age >40 years) with the development of LAHL. This was not seen in our cohort nor in other studies based in Southern Africa [16], [17], [18]. The majority of their subjects were from Europe and the Americas with an older age distribution than in our study (mean of 42 years for cases vs. 35 years).

For the subgroup of women with higher BMI, stavudine use, when controlling for other risk factors, remained associated with a significant increase in risk of LAHL. Switching these women to zidovudine conferred an 80% reduction in hazards of LAHL. These data suggest that higher-risk individuals should be switched off stavudine-based therapy in order to reduce adverse events.

There are several limitations to this study. The two treatment groups were quite different as demonstrated in Table 1. In our model we were able to control for the variables in the model, but not for unmeasured confounders (e.g. HIV clinical stage). Clinicians may have been more likely to test for hyperlactatemia in patients on stavudine resulting in a detection bias. However, 14% of subjects initiated on stavudine and 18% of subjects initiated on zidovudine had at least one serum lactate level checked during the study period, suggesting that serum lactate testing was not biased towards subjects on stavudine. We do not think deployment of the handheld lactate machine for screening (introduced in 2006) differentially affected case finding between the two groups. Any patient with a positive handheld device test required confirmatory serological testing; as above, testing rates were not higher for subjects initiated on stavudine. Our data, in combination with prior data evaluating risk factors for lactic acidosis and hyperlactatemia, strongly suggest that women and patients with higher BMI treated with stavudine are at high risk for developing LAHL. In addition, our data demonstrate that using zidovudine rather than stavudine, even among patients at highest risk for mitochondrial toxicities, dramatically reduces the risk of developing lactic acidosis. Further, for patients initiated on stavudine-based therapy, switching to zidovudine is protective. Although a recent Cochrane Review concluded there is no difference in treatment outcomes (toxicity, death, disease progression) for stavudine- compared with zidovudine-based ART, the randomized-controlled trials on which their analyses were based included patients from North America, the Caribbean, Australia and China [28]. However, most studies observing high rates of mitochondrial toxicities include patients from sub-Saharan Africa.

As of April 2010, first-line ART in South Africa includes tenofovir, lamivudine, and either efavirenz or nevirapine [29]. However, because of drug shortages many clinics remain unable to initiate all patients on tenofovir-based therapy and are not able to routinely switch patients to tenofovir-containing regimens. Thus, in South Africa (and other countries still using stavudine) these findings will be helpful in identifying patients who are at highest risk for stavudine-induced complications. While all patients will benefit from using alternatives to stavudine, avoiding this drug in women and patients with higher BMI may offer an effective and practical strategy for reducing the incidence of lactic acidosis and hyperlactatemia until countries can completely eliminate use of this agent.
